# Detection of dioxin-induced demethylation of mouse *Cyp1a1* gene promoter by a new labeling method for short DNA fragments possessing 5'-methylcytosine at the end

**DOI:** 10.1186/s41021-017-0089-5

**Published:** 2018-01-10

**Authors:** Hisaka Kurita, Toshiki Aiba, Toshiyuki Saito, Seiichiroh Ohsako

**Affiliations:** 1 Laboratory of Environmental Health Science, Center for Disease Biology and Integrative Medicine, The University of Tokyo, 7-3-1 Hongo, Bunkyo-ku, Tokyo, 113-8655 Japan; 2 Laboratory of Medical Therapeutics and Molecular Therapeutics, Gifu Pharmaceutical University, Daigaku-Nishi, Gifu, 501-1196 Japan; 3 Department of Radiation Effects Research, National Institutes for Quantum and Radiological Science and Technology, 4-9-1 Anagawa, Inage-ku, Chiba, 263-8555 Japan; 4 Center for Disease Biology and Integrative Medicine, Graduate School of Medicine, The University of Tokyo, 7-3-1 Hongo, Bunkyo-ku, Tokyo, 113-0033 Japan

**Keywords:** Restriction enzyme, Labeling, 5′-Methylcytosine, Methylome

## Abstract

Environmental factors stimulate alteration of DNA methylation level. Investigation of the genome-wide DNA methylation status is important for environmental health studies. We here designed a genomic DNA amplification and labeling protocol using a methylation-sensitive restriction enzyme *HinP1* I. This method can specifically amplify genomic DNA fragments possessing methyl-CpG at the end. The fragments are a relatively short size and dominantly located on CpG-islands. By using the samples prepared by this method, a dioxin-induced change in the methylation level of the mouse *Cyp1a1* promoter was successfully evaluated using oligonucleotide probes covalently bound onto a glass plate. The method developed in this paper would be useful for other genome-wide analysis platforms for the large scale epigenome-wide association studies (EWAS) including human epidemiological samples.

## Introduction

Epigenomic alterations induced by environmental factors such as endocrine-disrupting chemicals are interested by many researchers [1–3]. Analysis for methyl-CpG frequency is preferentially performed because genomic DNA is stable and easily stored in a laboratory as a batch of sample stocks. If an appropriate amplification method is possible, such as methylation-sensitive PCR, even a minute amount of genomic DNA is sufficient. Epigenome-wide association studies (EWAS) were introduced as a new concept [4]. Methyl-sensitive restriction enzymes are generally employed in EWAS. *Hpa* II tiny fragment enrichment by ligation-mediated PCR (HELP) and microarray-based integrated analysis of methylation (MIAM) are used in many studies [5–8]. However, these methods have some difficulties in sample preparation. Usage of a rare restriction cutter in HELP analysis results in the decrease in the detectable number of methyl-CpGs. In MIAM, two reaction tubes has to be prepared for two restriction enzymes, that is, methylation-sensitive *Hpa* II and the nonsensitive isoschizomer *Msp* I. Thus, alternative restriction-enzyme based sample preparation and simple labeling methods is still being desired especially for analysis of multiple samples [9, 10].

Most recently, we have reported on methylated site display-amplified fragment length polymorphism (MSD-AFLP) analysis as a new, sensitive, and affordable method of genome-wide CpG methylation analysis [11]. This novel method is based on a new concept, that is, methylation site display (MSD). MSD specifically amplifies DNA fragments with methyl-CpG at one end (*Hpa* II) the eight-nucleotide recognition restriction enzyme *Sfb* I site at the other end. MSD-AFLP was designed not only for mouse genome but also for human genome [11], because the human methylome analysis using large scale clinical samples is expected. Since high resolution is required in the next step of amplified fragment length polymorphism (AFLP) analysis, this labeling method amplifies relatively long DNA fragments. Therefore, MSD-AFLP preferentially detects methyl-CpGs located on non-CpG islands.

In this report, a protocol for DNA amplification and labeling is presented to improve the probability of the detection of methyl-CpGs located preferentially in CpG islands. We have already reported the demethylation of a CpG in the mouse *Cyp1a1* promoter by dioxin exposure [12, 13]. Using methylation sensitive-restriction enzyme-dependent-PCR (MSRE-PCR) with the mouse liver genomic DNAs, we found three fold of change in 5-methylcytosine frequency [13]. Here, we prepared the postnatal days 14 mouse liver DNAs which was exposed to TCDD on gestational stage. We used these samples to examine if this method can detect a reduction of CpG methylation of cytochrome P450 1a1 (*Cyp1a1*) gene promoter DNA by using glass array, to verify the effectiveness of our new protocol.

## Methods

### Reagents

2,3,7,8-Tetrachlorodibenzo-*p*-dioxin (TCDD) was purchased from Cambridge Isotope Laboratory (Andover, MA, USA). Wizard Genomic DNA Purification kit and pGEM-T Easy Vector used in this study were from Promega (Madison, WI, USA). Oligonucleotides purified by HPLC were all purchased from Hokkaido System Science Co., Ltd. (Sapporo, Hokkaido, Japan). Restriction enzymes *Nco* I and *Xba* I were from Toyobo (Kita-ku, Osaka, Japan). Restriction enzyme *HinP1* I was from New England BioLabs Inc. (Ipswich, MA, USA). Ligation Convenience kit was from Nippon Gene (Chiyoda-ku, Tokyo, Japan). PCR Purification kit and QIAquick Gel Extraction kit were from QIAGEN (Hilden, Germany). TaKaRa Ex Taq polymerase, RNA Transcript SureLABEL™ Core Kit, Solution I, and TaKaRa Hubble Slide Glass were from TaKaRa Bio (Kusatsu, Shiga, Japan). Big Dye Terminator v3.1 Cycle Sequencing kit was from ThermoFisher Scientific Inc. (San Diego, CA, USA). Cy3-UTP or Cy5-UTP were purchased from GE Healthcare UK Ltd. (Buckinghamshire, UK). OpHyb Hybridization buffer kit was from Operon Technologies (Alameda, CA, USA). LightCycler 480 SYBR Green I Master was from Roche Diagnostics Japan (Minato-ku, Tokyo, Japan).

### Animals and treatments

The animal experimentation protocols of this study were reviewed and approved by the Animal Care and Use Committee, The University of Tokyo. Pregnant C57BL/6 J mice were from Japan CLEA (CLEA Japan, Inc. Tokyo, Japan). They were orally administered TCDD (3 μg/kg bw) or vehicle control (corn oil) on gestational days 12.5. One of them was sacrificed on postnatal day 14 to collect liver samples. Control liver samples were also collected from an untreated pregnant mouse. Genomic DNA was isolated by Wizard Genomic DNA Purification kit.

### Methyl-CpG-DNA short fragment amplification

**Fig. 1:**
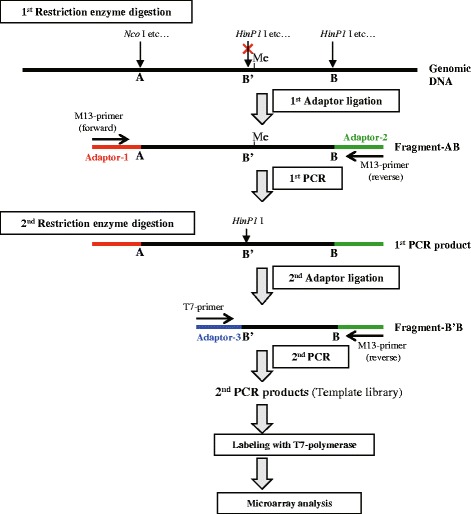
Flowchart of sample preparation for the amplification of methyl-CpG DNA fragments and labeling. The first restriction enzyme digestion is performed with *Nco* I (alternatively, other 5′-, or 3′-overhanging sticky-end restriction enzymes can be used) and *HinP1* I (alternatively, other methyl-sensitive sticky-end restriction enzymes, such as *Hha* I, *Hpa* II can be used). If the B-*HinP1* I site is unmethylated and the B′-*HinP1* I site is methylated, the resulting Fragment-AB will be amplified in by the first PCR after the first ligation with *Adaptor-1* and *Adaptor-2*. By the second digestion of the first PCR product with restriction enzyme *HinP1* I, the amplified the Fragment-AB will be cut to form the smaller Fragment-B’B. After ligation with *Adaptor-3*, the Fragment-B’B will be amplified in the second PCR, which means that the B′-*HinP1* I site is methylated in genomic DNA. In the case of the B′-unmethylated state, the Fragment-AB’ is amplified in the first PCR, but the Fragment-B’B is not amplified in the second PCR at all

Figure 1 represents the flow of the DNA preparation protocol developed in this study. The oligonucleotides used for adaptors and the primers used for PCR reaction are shown in Table 1. Liver genomic DNA (100 ng) was digested with *Nco* I and the methyl-sensitive restriction enzyme *HinP1* I in 20 μL reaction volume (1st Restriction enzyme digestion). After DNA purification with the PCR Purification kit, 100 pM *Adaptor-1* containing the M13 forward sequence and *Adaptor-2* containing the M13 reverse sequence were ligated at room temperature for 1 h in 100 μL reaction volume (1st Adaptor ligation). After DNA purification, the sample was subjected to PCR with *M13 forward* and *M13 reverse* primers and TaKaRa Ex Taq polymerase in 100 μL reaction volume using the following program; initial denaturation at 94 °C for 2 min; denaturation at 94 °C for 30 s; annealing at 52 °C for 45 s; extension at 72 °C for 5 min; 5 cycles (1st PCR). The purified PCR product was then digested again only with *HinP1* I in 20 μL reaction volume (2nd Restriction enzyme digestion). After DNA purification, 100 pM *Adaptor-3* containing T7 promoter sequence was ligated at room temperature for one hour in 100 μL reaction volume (2nd Adaptor ligation). After DNA purification, the sample was subjected to PCR with the *T7* primer and *M13 reverse* primer in 100 μL reaction volume using the following program; initial denaturation at 94 °C for 2 min; denaturation at 94 °C for 30 s; annealing at 52 °C for 45 s; extension at 72 °C for 5 min; 25 cycles (2nd PCR). After the purification, the 2nd PCR products were used as DNA template library.

**Table 1:** Oligonucleotides used in this study

Oligonucleotide-name		Sequences (5′ to 3′)
Oligonucleotides for adaptors		
*Adaptor-1*	Upper	TCACGACGTTGTAAAACGACGGCCAGC
	Lower	p-CATGGCTGGCCGTCGTTTTACAACGTCGTGA
*Adaptor-2*	Upper	p-CGAGTCATAGCTGTTTCCTGTGTGAAATTGTTCCGC
	Lower	GCGGAACAATTTCACACAGGAAACAGCTATGACT
*Adaptor-3*	Upper	TGAATTGTAATACGACTCACTATAGGGG
	Lower	p-CGCCCCTATAGTGAGTCGTATTACAATTCA
Primers for amplification		
*M13 forward*		GTAAAACGACGGCCAG
*M13 reverse*		CAGGAAACAGCTATGAC
*T7*		TAATACGACTCACTATAGGG
*Sp6*		ATTTAGGTGACACTATAGAA
Gene-specific primers		
*T7 + Cyp1a1*		TGAATTGTAATACGACTCACTATAGGGGCGCAACGA
*M13 + Cyp1a1*		CAGGAAACAGCTATGACTCGCCACTGGC
*Rat E-cadherin forward*		ATGGGAGCCCGGTGCCGCAGCTT
*Rat E-cadherin reverse*		CTCTGTGGTGATGCCGGTGGTGG
Oligonucleotides for custom-made glass array		
*Mouse Cyp1a1 oligo*		a-CGCCACTGGCCTTCCTGTCCTGTGACCTCT
*Rat E-cadherin oligo*		a-TGGCCCAGGGACTTCAGTGTCACTTTGGTA
Primers for MSRE-PCR		
*Cyp1a1 forward*		TTCCTGTCCTGTGACCTCTG
*Cyp1a1 reverse*		TTGCACCCCTGAAACATTCA

p: 5′-phosphorylation; a: 5′-amination

### In vitro transcription

For converting to the DNA template library to fluorescent labeled amplified RNA (aRNA), the RNA Transcript SureLABEL™ Core Kit with Cy3-UTP or Cy5-UTP according to the manufacturer’s instruction.

### Amplification with gene-specific primers

Gene-specific primers for the mouse *Cyp1a1* promoter DNA were constructed by connecting the T7 sequence to the 9-bp *Cyp1a1* promoter sequence (*T7 + Cyp1a1*) and the M13 reverse sequence to the 10-bp *Cyp1a1* promoter sequence (*M13 + Cyp1a1*) (Table 1). These oligonucleotides were used in PCR using the following program, initial denaturation at 94 °C for 2 min; denaturation at 94 °C for 15 s; annealing at 52 °C for 10 s; extension at 72 °C for 15 s; 30 cycles. The PCR products were then subjected to 5% agarose gel electrophoresis. Thick bands around 100 bp were purified with QIAquick Gel Extraction kit, subcloned to pGEM-T Easy Vector, and then sequenced by using Big Dye Terminator v3.1 Cycle Sequencing kit.

### Custom glass array

The oligonucleotide probes of 100 μM *Mouse Cyp1a1 oligo* and *Rat E-cadherin oligo* were diluted to 25 μM with Solution I and then spotted on TaKaRa Hubble Slide Glass using DNA Manual Arrayer (Greiner Inc., Germany). After air-drying, the slide was incubated in 0.2% SDS for 2 min, distilled water for 30 s twice, 0.3 N NaOH for 5 min, distilled water for 30 s twice, boiled water for 2 min, and 100% ethanol for 3 min at 4 °C, and then air-dried to fix the oligonucleotides on the slide glass.

### Competitive hybridization of fluorescence labeled aRNAs

For preparation of labeled RNA as external control, we cloned rat E-cadherin cDNA using RT-PCR with rat testicular total mRNA, subcloned it into pGEM vector, and subjected to PCR with *T7* and *Sp6* primers to make E-cadherin DNA template. This E-cadherin template was transcribed with Cy3- or Cy5-UTP to prepare external control aRNAs. The Cy3-aRNA (6 μg) transcribed from the DNA template library of control mouse liver and the Cy5-aRNA (6 μg) from those of the TCDD-treated mouse liver described above were mixed with Cy3-E-cadherin aRNA (0.4 μg) and with Cy5-cadherin aRNAs (0.4 μg), respectively. Equal volume of two mixtures were mixed and then competitively hybridized by using OpHyb Hybridization buffer kit to E-cadherin and mouse *Cyp1a1* promoter oligonucleotides probes on the hand-made glass array described above. After washing, the array was scanned using GenePix Personal 4100b (Axon Instruments, Sunnyvale, CA, USA) to measure Cy3 or Cy5 spot intensities. Relative fluorescence intensity representing −499-CpG methylation level was calculated to divide the mean of *Cyp1a1* promoter spot intensity (3 spots) by the mean of E-cadherin spot intensity (3 spots) of each dye. Three independent data from three blocks were used in the statistical analysis.

### MSRE-PCR

Methylation frequency of mouse *Cyp1a1* promoter region at −499 (−499-CpG) was determined by MSRE-PCR described in our previous study [13]. Primer sequences used were in Table 1. Briefly, purified genomic DNA (100 ng) was divided into two portions. One aliquot was digested with methylation-sensitive *HinP1* I while the other aliquot was digested with *Xba* I. *HinP1* I-digested and *Xba* I-digested DNA were subjected to the quantitative PCR using the LightCycler® 480. The methylation level was represented as a ratio of target copy numbers from the *HinP1* I-digested DNA versus those from the *Xba* I-digested DNA.

## Results and discussion

**Fig. 2:**
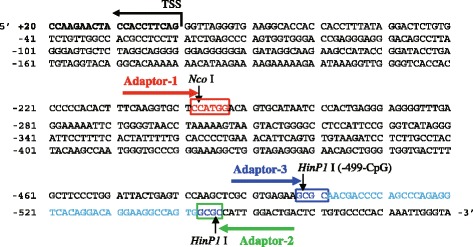
Representation of mouse *Cyp1a1* gene promoter region. The sequence is presented as reverse complement (5′ to 3′ end). Methyl-CpG at −499 from transcription start site (TSS) was indicated in *HinP1* I site (blue, B′ in Fig 1). Another *HinP1* I site and *Nco* I site were indicated as green (B in Fig. 1) and red (A in Fig. 1), respectively. The −499-CpG was 30% methylated in the control mouse liver genome DNA in our previous study [13]. The 45-bp sequence indicated by light blue character was amplified by the *T7* primer and *M13* reverse primer, which contain two adaptor sequences, *Adaptor-3* and *Adaptor-2*, respectively. The PCR product containing this sequence was detected as an approximately 100-bp in Fig. 3a

**Fig. 3:**
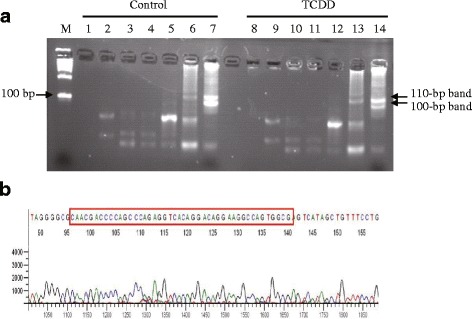
Electrophoretic patterns in the step-wise and sequencing of PCR product. **a** Lanes 1 and 8, just after the 1st Restriction enzyme digestion (*HinP1* I and *Nco* I); lanes 2 and 9, after the 1st Adaptor ligation (*Adaptor-1* and *Adaptor-2*); lanes 3 and 10, after the 1st PCR (*M13 forward* and *M13 reverse* primers); lanes 4 and 11, after the 2nd Restriction enzyme digestion (*HinP1* I); lanes 5 and 12, after the 2nd Adaptor ligation (*Adaptor-3*); lanes 6 and 13, after the 2nd PCR as amplified methyl-CpG DNAs (*T7* and *M13 reverse*); lanes 7 and 14, the products after PCR with gene-specific primers (*T7 + Cyp1a1* and *M13 + Cyp1a1*). Around 100-bp position, two thick bands (100 bp and 110 bp) were detected after PCR with gene-specific primers (lanes 7 and 14). **b** Sequence result of 100-bp band. The sequence was matched with mouse *Cyp1a1* promoter region indicted as 45-bp light blue characters in Fig. 2

In the present study, we analyzed the liver DNAs of 14 days old mice which was exposed prenatally to TCDD. A cytosine residue of the *HinP1* I site (−499-CpG) in the mouse *Cyp1a1* in the promoter region presented in Fig. 2 as a blue square is methylated by approximately 30% in the normal mice liver, but its level decreased to less than 10% in case of the TCDD-treated adult mice [13]. The 45-bp DNA sequence is expected to be amplified from the 2nd PCR products (blue, Fig. 2). In order to validate the quality of the 2nd PCR products (Template library), we further amplified the target fragment containing 45-bp Cyp1a1 gene using gene-specific primers. After electrophoresis, at the approximately 100-bp position, two thick bands (100 bp and 110 bp) were detected (Fig. 3a, lanes 7 and 14). We isolated these two bands and subcloned them into pGEM vectors followed by sequencing. The 100-bp lower band was revealed to have the expected *Cyp1a1* promoter region sequence (Fig. 3b). Although the 110-bp band showed no significant difference in intensity between the control (lane 7) and the TCDD-treated (lane 14) samples, the 100-bp band of control DNA (lane 7) was more intense than that of TCDD (lane 14), indicating that the content of the target *Cyp1a1* DNA fragment in the control sample was higher than that in the TCDD-treated sample. This finding suggests that our protocol can more efficiently amplify methyl-DNA targets. The 110-bp band was revealed to contain a portion of the sequence in chromosome 11 genomic contig (C57BL/6 J, NT_039515) by BLAST search. The DNA sequence also has two *HinP1* I sites as expected and the *Nco* I site close to these *HinP1* I sites. The reason for the nonspecific amplification of this 110-bp band seems to be the sequence similarity with one mismatch to the gene-specific primers we designed.

**Fig. 4:**
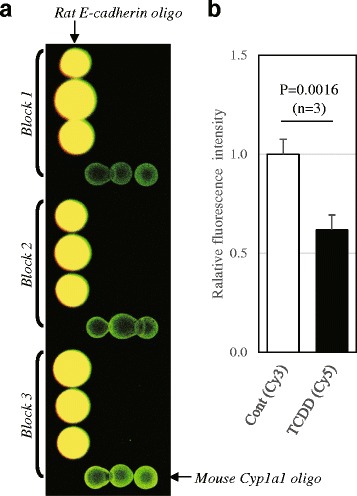
Competitive hybridization using custom-glass array for comparison of −499-CpG methylation level between control and TCDD-treated mouse liver DNAs. **a** Glass array image of the merged Cy3 and Cy5 fluorescence. Three spots of *Rat E-cadherin oligo* and *Mouse Cyp1a1 oligo* were set on one block. Using the new method in this study, Cy3- and Cy5-labeled aRNAs were produced using control and TCDD-treated mouse liver DNAs, respectively. They were then competitively hybridized on the custom glass array. The picture represents three independent blocks. Image and each spot fluorescence was obtained by GenePix instrument. Note that the spots of *Rat E-cadherin oligo* spot showed yellow whereas the spots of *Mouse Cyp1a1 oligo* showed green, indicating −499-CpG methylation level of control mouse is higher than that of TCDD-treated mouse. **b** Comparison of fluorescence intensity between control (Cy3) and TCDD-treated mice (Cy5). Averages of relative spot fluorescence intensity of *Mouse Cyp1a1 oligo* of each dye were calculated as described in Materials and Methods. Relative fluorescence intensity representing −499-CpG methylation level was calculated to divide the mean of *Mouse Cyp1a1 oligo* spot intensity (arrowed 3 spots, in A) by the mean of *Rat-E-cadherin* spot intensity (arrowed 3 spots, in A) of each dye. Three data (Block1 to 3) were used in the statistical analysis (Student’s t-test)

**Fig. 5:**
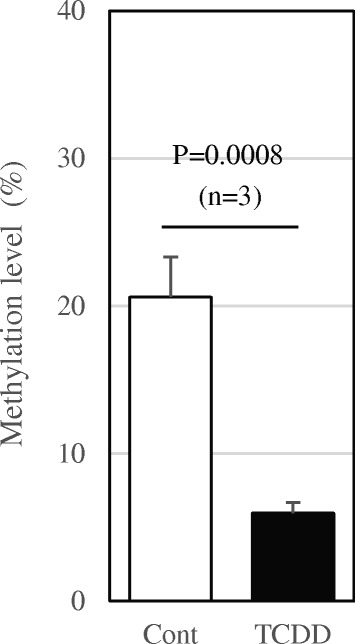
MSRE-PCR analysis for −499-CpG methylation. Mouse genomic DNAs from control and TCDD-treated mice (*n* = 3) were digested *HinP1* I and then subjected to quantitative MSRE-PCR to measure the % methylation level at −499-CpG as described in Materials and Methods. Statistical significance was analyzed by Student’s t-test

Next, we constructed Cy3-UTP- or Cy5-UTP-labeled fluorescent aRNA probes using the 2nd PCR products as the templates by T7 in vitro transcription. The amplified methyl-CpG DNAs from the control and TCDD-treated samples were labeled with Cy3-UTP and Cy5-UTP, respectively. These two labeled probes were then competitively hybridized to the hand-made glass array spotted with the mouse *Cyp1a1* promoter region oligonucleotides and those of rat E-cadherin as the external control (Fig. 4a). Expectedly, the Cy3 signal in *Cyp1a1* was approximately two fold stronger than the Cy5 signal. The calculated average signal ratio (Cy5/Cy3) was 0.618 ± 0.04. To confirm this result, MSRE-PCR analysis was performed directly with genomic DNAs. The −499-CpG methylation level was reduced to 28.9% by TCDD exposure (Fig. 5). From these results, the method in this study was demonstrated to efficiently detect methylation level of CpG sites (Fig. 4b).

This method appears to preferentially amplify relatively short DNA fragments (*HinP1* I-*HinP1* I) that are methylated at the 5’end, probably located in CpG islands. *HinP1* I (G_ˇ_CGC) sites as well as other methylation-sensitive restriction enzymes, such as *Hpa* II (C_ˇ_CGG), *Hha* I (GCG_ˇ_C), and *BstU* I (CG_ˇ_CG), are clustered in CpG islands. It has been reported that by using the 21 chromosomes of the mouse genome assembly (mm10, GRCm38, Dec2011 build UCSC), in silico prediction of enzymatically digested fragments revealed that *Hpa* II site and *Hha* I sites covered the most UCSC-annotated CpG island with 94.8 and 93.4%, respectively [14]. This indicates that the short *HinP1* I-*HinP1* I DNA fragments should be predominantly from in CpG islands. The assay using the Illumina Infinium HumanMethylation480 or MethylationEPIC BeadChips has recently been mentioned as the most cost-effective method and used for large-population studies [15]. This platform is based on sodium bisulfite treatment and subsequent microarray analysis. However, the main reason for this widespread utilization is the design of the Infinium BeadChip platform which has bias towards 480 K CpG sites and covers 96% CpG islands [16].

The fluorescently labeled aRNA probes generated from DNA sources after the 2nd PCR (Template library) are very useful for many applications including genome-wide tiling array analysis to determine CpG methylation levels [17]. In addition to microarray analysis, this amplification method can be applied to the sequencing-based analyses [18]. In this study, we used only *Nco* I as the proximal restriction site for the first PCR. Coverage of CpGs will increases by using as many as six-nucleotide recognition restriction enzymes in the first step as possible. Then analysis will be more comprehensive for detecting methyl-CpG sites in genome-wide. The expected coverage and power will be similar to those of the currently used method, that is, the methylation-sensitive restriction enzyme-based sequencing method (MRE-seq) [19].

Amplification protocols using methylation-sensitive restriction enzymes, such as HELP have to amplify non-methylated DNAs in an extra control reaction tube [6, 8]. Unlike these current protocols, our method here amplifies and detects only methyl-CpG DNAs in a single reaction tube; therefore, the method is suitable for the simultaneous analysis of multiple samples. Because of this great advantages, the amplification and labeling method presented in this study will be useful in large-scale epidemiological studies using human samples as well as MSD-AFLP [20, 21].

## Abbreviations

aRNA: Amplified RNA
Cyp1a1: Cytochrome P450 1a1
EWAS: Epigenome-wide association studies
MSD-AFLP: Methylated site display-amplified fragment length polymorphism
MSRE-PCR: Methylation-sensitive restriction enzyme-dependent PCR
TCDD: 2,3,7,8-Tetrachlorodibenzo-p-dioxin
